# Preparation of Hollow Polyaniline Micro/Nanospheres and Their Removal Capacity of Cr (VI) from Wastewater

**DOI:** 10.1186/s11671-018-2815-8

**Published:** 2018-12-07

**Authors:** Honge Wu, Qing Wang, Guang Tao Fei, Shao Hui Xu, Xiao Guo, Li De Zhang

**Affiliations:** 1 Key Laboratory of Materials Physics and Anhui Key Laboratory of Nanomaterials and Nanotechnology, Institute of Solid State Physics, Hefei Institutes of Physical Science, Chinese Academy of Sciences, P. O. Box 1129, Hefei, 230031 People’s Republic of China; 2 University of Science and Technology of China, Hefei, 230026 People’s Republic of China; 3 College of Biological and Chemical Engineering, Anhui Polytechnic University, Wuhu, 241000 People’s Republic of China

**Keywords:** Reproducible, Toxic Cr (VI), Removal, Wastewater, Hollow polyaniline micro/nanospheres

## Abstract

The hollow polyaniline (PANI) micro/nanospheres are obtained through a simple monomer polymerization in alkaline solution with Triton X-100 Micelles as soft templates. The hollow PANI micro/nanospheres demonstrate rapid and effective removal ability for Chromium (VI) (Cr (VI)) in a wide pH range, and the maximum removal capacity can reach 127.88 mg/g at pH 3. After treated with acid, the used hollow PANI micro/nanospheres have about the similar removal capacity of Cr (VI) from wastewater.

## Background

Heavy metal ion Chromium (VI) (Cr (VI)), originating from chromium plating, leather tanning, metal finishing, and textile industries, is seriously hazardous to ecosystems and living organisms due to its carcinogenicity and mobility [1–3]. The untreated Cr (VI) ions can cause kidney failure, pulmonary congestion, gastric damage, liver cancer, skin irritation, and so on [4–6]. In comparison to Cr (VI), Cr (III) ions are easily precipitated or adsorbed [7–9]. Thus, converting Cr (VI) to Cr (III) and precipitating Cr (III) to solid are the common techniques for Cr (VI) removal from solution. Whereas the traditional reductants such as sulfur dioxide, sodium metabisulfite, and ferrous sulfate, used for Cr (VI) reduction, are non-recyclable and not reusable. Additionally, they also lead to secondary waste products generation, leading to increased environmental problems [10, 11]. Therefore, it is essential to explore new materials for removal of Cr (VI) from aqueous environment.

Since Rajeshwar and co-workers firstly reported that conductive polymers could convert highly toxic Cr (VI) to less toxic Cr (III) in 1993 [12], conductive polymers, especially polyaniline (PANI), have been widely concerned [13–16], due to their easy synthesis, low cost, and special proton doping/dedoping mechanism. PANI, normally has three oxidation states, which are pernigraniline (PB, which belongs to aromatic tertiary amine), emeraldine (EB, which belongs to aromatic secondary amine), and leucoemeraldine (LB) [17, 18], contains benzenoid and quinonoid units with abundant amine groups which can provide electrons for Cr (VI) reduction [1, 10]. However, PANI bulk and film with poor porosity restricts its application in Cr (VI) reduction. Recently, the hollow PANI micro/nanospheres have received considerable attention due to their wide potential applications in supercapacitor [19, 20], electrochemical biosensors fields [21], and so on. Moreover, the hollow PANI micro/nanospheres with inner cavity can enhance specific surface area thus to enhance removal of Cr (VI) capacity and absorption rate. Recently, hollow PANI microspheres are being prepared using hard template methods [19]; however, they involve complex procedures of preparing and removing, which lead to poor reproducibility and make it rather difficult to retain the hollow structure after template removal [22, 23]. Compared to the hard template methods, the soft template methods are cheaper and more effective. More importantly, the soft template can be removed easily by water and ethanol [1, 24]. Hence, a simple, effective, and high-yield preparation method of PANI micro/nanospheres is still desirable in the field of toxic Cr (VI) removal.

In this paper, a large-quantity of reproducible hollow PANI micro/nanospheres is synthesized by a simple monomer polymerization in alkaline solution with Triton X-100 Micelles as soft templates. The reproducible hollow PANI micro/nanospheres are nontoxic and safe to ecosystems. Meanwhile, the reproducible hollow PANI micro/nanospheres have high removal of Cr (VI) capacity which can reached 127.88 mg/g at pH 3. The removal Cr (VI) kinetics model and absorption isotherm of hollow PANI micro/nanospheres conform to pseudo-second-order kinetics model and Langmuir absorption isotherm model, respectively. The hollow PANI micro/nanospheres not only remove Cr (VI) rapidly but also can be easily regenerated for reuse.

## Methods

### The Aim of the Study

To solve the problem which the heavy metal ion Cr (VI) in wastewater brings a lethal hazardous to ecosystems and living organisms, the reproducible hollow PANI micro/nanospheres are prepared through a simple monomer polymerization in alkaline solution with Triton X-100 Micelles as soft templates, in order to remove Cr (VI)-bearing waste.

### Materials

Aniline (Sinopharm Co. Ltd), sodium hydroxide (NaOH, Sinopharm Co. Ltd), Triton X-100 (Alfa), and ammonium persulfate (APS, Sinopharm Co. Ltd) are analytical grade and used without further purification.

### Synthesis of Hollow PANI Micro/Nanospheres

Hollow PANI micro/nanospheres were prepared by a simple polymerization of monomer in alkaline solution with Triton X-100 Micelles as templates. In a typical synthesis process, 32 mmol aniline, 32 mmol NaOH, and 0.82 mmol Triton X-100 were directly dispersed in 20 mL deionized water with a magnetic stirring at room temperature for 20 min, then the mixture solution was cooled in the ice-water bath for 5 min. After that, the oxidant aqueous solution (20 mL) containing 32 mmol APS precooled in the ice-water bath for 5 min was added to the above-mentioned aniline mixture solution in one portion, and the resulting solution was stirred for another 0.5 min to ensure complete mixing and then the reaction was proceed in the ice-water bath without agitation for 12 h. Finally, the products were washed and centrifuged with deionized water and ethanol until the filtrate became colorless and then dried in an oven for 24 h at 60 °C.

### Characterization

The morphology of the resulting PANI products was observed with field-emission scanning electron microscope (FESEM, Sirion 200) and transmission electron microscope (TEM, JEOL-2010). The structures of the as-prepared PANI were characterized by X-ray diffraction (XRD, Philips X’Pert) and Fourier-transform infrared spectroscopy (FTIR, JASCO FT-IR 410 spectrophotometer). The Cr concentration was analyzed by inductively coupled plasma emission spectrometer (ICP) and UV-Vis absorption spectroscopy. The oxidation state of chromium adsorbed on PANI nanostructure was ascertained using X-ray photoelectron spectroscopy (XPS, ESCALAB 250). The zeta potential and particle size were obtained by Zetasizer 3000HSa.

### Removal of Cr (VI) by Hollow PANI Spheres

The Cr (VI) solution was prepared by dissolving potassium dichromate (K_2_Cr_2_O_7_) into deionized water. The stock solution (2 mmol L^−1^) was prepared by dissolving 1.177 g of K_2_Cr_2_O_7_ in 2000 mL of deionized water. All working solutions with various concentrations were obtained by successive dilution. The as-synthesized PANI powder (10 mg) was ultrasonically dispersed into 20 mL of Cr (VI) solution (1.2 mmol L^−1^) with different pH values, which was adjusted with NaOH and HCl solution. After reaction for 3 h, the reaction solution was centrifuged and the pH values of supernatant liquid were adjusted into the range of 7.5–8.5. Finally, the used hollow PANI micro/nanospheres was separated and rinsed several times with deionized water and dried. The Cr (VI) removal capacities of the hollow PANI micro/nanospheres in different pH value can be calculated using the following equation:

1 $$ {q}_e=\frac{\left({c}_0-{c}_e\right)V}{m} $$

where *q*_e_ is the amount of Cr (VI) removal per gram of hollow PANI micro/nanospheres at equilibrium (mg/g), *V* is the volume of the solution (L), *m* is the mass of the hollow PANI micro/nanospheres (g), and *c*_0_ and *c*_e_ are the concentration of the Cr (VI) at initial and equilibrium (mg/L), respectively.

### Removal Kinetics Measurement

For the experiments of removal kinetics, three sets of experiments were carried out, in which the pH values of Cr (VI) solution were 3, 4, and 5, respectively. The as-synthesized PANI powder (10 mg) was ultrasonically dispersed into 20 mL of Cr (VI) solution (1.2 mmol L^−1^) with different pH values, and the mixture was stirred magnetically. At predetermined intervals, an appropriate amount of the reaction solution was taken out and then centrifuged. The supernatant liquid was used for analyzing the Cr (VI) concentration by UV-Vis absorption spectroscopy at 350 nm wavelength. The theoretical kinetic plots were obtained using the pseudo-second-order equation:

2 $$ \frac{t}{q_t}=\frac{1}{k_2{q}_e^2}+\frac{t}{q_e} $$

where *q*_e_ and *q*_t_ are the amounts of Cr (VI) removed by hollow PANI micro/nanospheres at equilibrium and at time *t* (min) (mg/g), and *k*_2_ is the rate constant (g/mg min).

### Removal Isotherm Measurement

For the removal isotherm experiments, the operation processes are the same as above. We also did three sets of experiments in different pH values, 3, 4, and 5, respectively. In each experiment, the as-synthesized PANI powder (10 mg) was ultrasonically dispersed into 20 mL of Cr (VI) solution with different concentration under magnetic stirring. When the solution reached to the equilibrium of removal, they were centrifuged and then analyzed for the remaining Cr (VI) concentration by UV-Vis absorption spectroscopy. The Langmuir plots for the removal of Cr (VI) by hollow PANI micro/nanospheres using the Langmuir equation:

3 $$ \frac{c_e}{q_e}=\frac{1}{q_m{k}_L}+\frac{c_e}{q_m} $$

where *q*_m_ is the maximum removal capacity (mg/g), *q*_e_ is the amounts of Cr (VI) at equilibrium (mg/g), *c*_e_ is the concentration of the Cr (VI) at equilibrium (mg/L), and *k*_L_ is the Langmuir constant.

## Results and Discussion

**Fig. 1:**
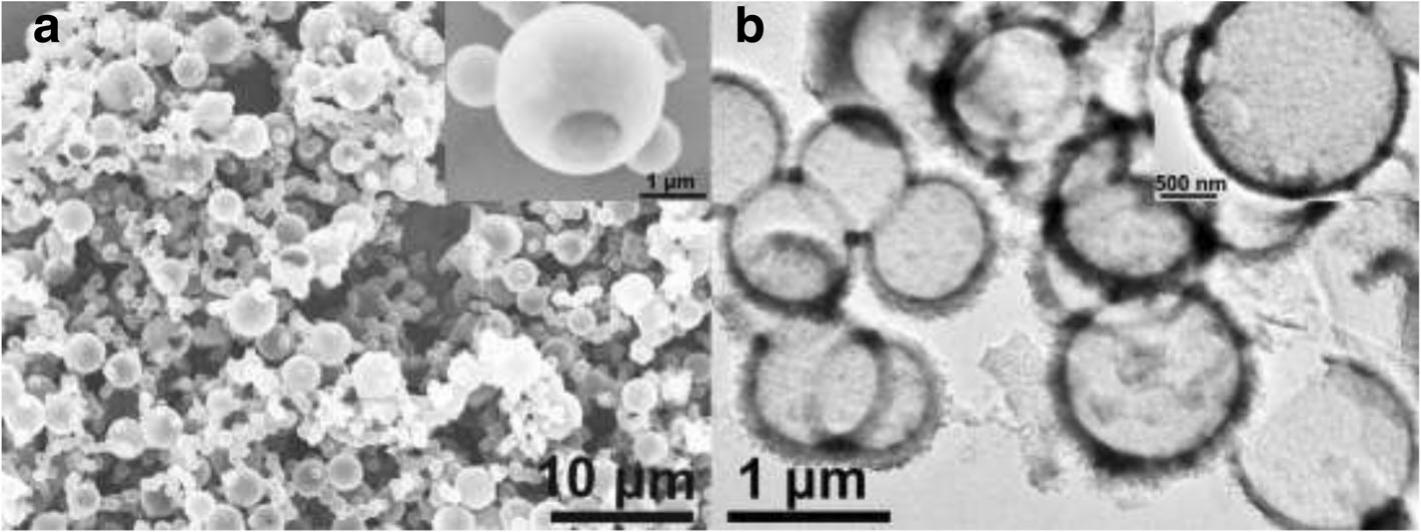
**a** SEM and **b** TEM images of the as-synthesized hollow PANI micro/nanospheres

The as-synthesized PANI was obtained by monomer polymerization with Triton X-100 Micelles as soft templates. The morphology via SEM and TEM observation is shown as Fig. 1. Abundant PANI micro/nanospheres can be observed clearly from SEM image of PANI in Fig. 1a. Carefully, a hole on the micro/nanospheres surface is observed, as shown in the inset image of Fig. 1a, which indicates the micro/nanospheres are hollow. This result is further confirmed via TEM image (Fig. 1b). In addition, two images show that the diameter of these micro/nanospheres is between 0.5 and 2 μm.

**Fig. 2:**
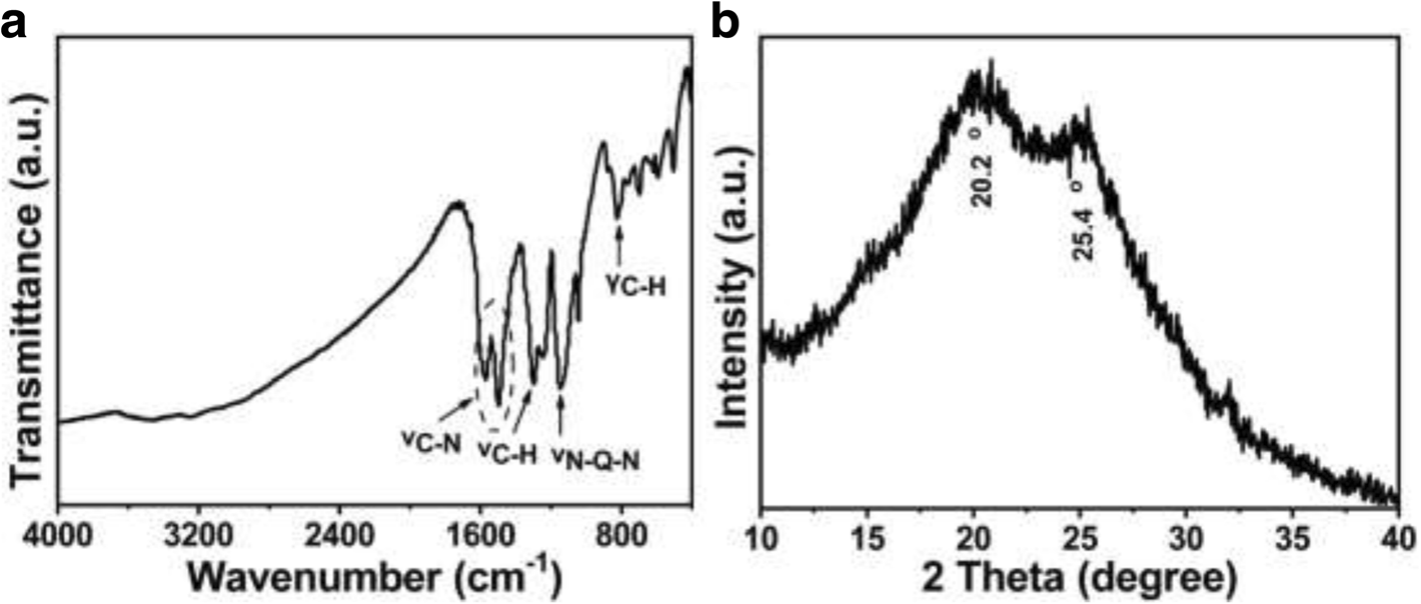
**a** FTIR spectrum and **b** XRD patterns of the as-synthesized hollow PANI micro/nanospheres

The molecular structure of as-synthesized hollow PANI micro/nanospheres is characterized by FTIR spectroscopy and X-ray diffraction (XRD), shown in Fig. 2. Five characteristic peaks can be observed clearly in Fig. 2a. The characteristic peaks at 1569 cm^−1^ and 1496 cm^−1^ are attributed to the C-N stretching vibration of quinoid ring (Q) and benzenoid ring (B), respectively. The peak appeared at 1298 cm^−1^ is due to the C-H stretching vibration with aromatic conjugation [1]. Peak at 1142 cm^−1^ corresponds to the N-Q-N stretching modes and is a symbol of electron delocalization in PANI [1]. Besides, the absorption peak at 824 cm^−1^ is characteristic of the C-H out-of-plane bending vibrations of the para-substituted benzene ring [25]. Comparing the characteristic peaks of the benzenoid ring and the quinoid ring, the relative absorbance intensity of benzenoid ring is greater than that of quinoid ring. Then, it can be deduced that the hollow PANI micro/nanospheres are mainly in emeraldine form. Figure 2b displays the XRD pattern of as-synthesized hollow PANI micro/nanospheres, which shows unusual high crystallinity with diffraction peaks centered at 20.2° and 25.4°, corresponding to the periodicity parallel and perpendicular to the polymer chains [25].

**Fig. 3:**
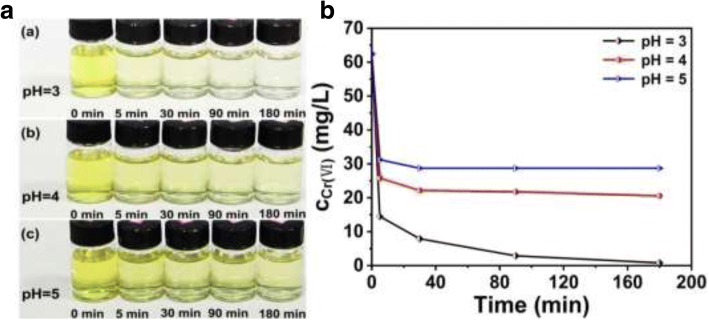
The Cr (VI) solution after treated with hollow PANI micro/nanospheres in different times, solution pH respectively at **a** 3, **b** 4, and **c** 5 (initial Cr (VI) concentration: 1.2 mmol/L (62.4 mg/L)). **a** The photographs and **b** Cr (VI) concentration in the final solution

Figure 3 shows the results of Cr (VI) removal at different times, in which the pH values of Cr (VI) solution are 3, 4, and 5, respectively. Seen from the Fig. 3a, the color of Cr (VI) solution is getting lighter under treatment with hollow PANI micro/nanospheres as increasing time. Especially, the relatively clear liquid is obtained after 90 min when the pH is 3. And the results which the concentration of Cr (VI) was treated by hollow PANI micro/nanospheres are directly shown in Fig. 3b. These results indicate that the hollow PANI micro/nanospheres are an efficient candidate for Cr (VI) removal from solution in appropriate condition.

**Fig. 4:**
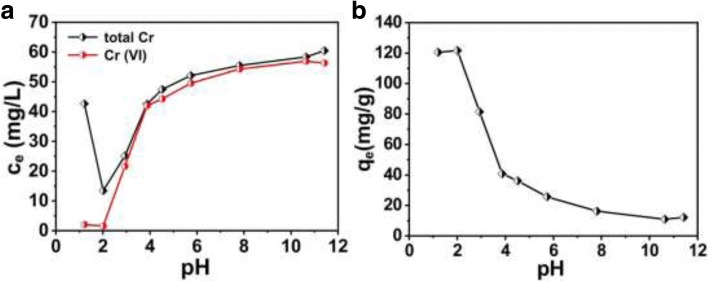
**a** The changes of the Cr concentration under different solution pH, and **b** the corresponding Cr (VI) removal capacities (initial Cr (VI) concentration: 1.2 mmol/L (62.4 mg/L))

The pH value of the solution is an important parameter which affects the chemistry properties of Cr (VI) in solution or the protonation or deprotonation of the PANI. Therefore, the Cr (VI) removal and reduction capacities of as-synthesized PANI are studied in solution of different pH, as shown in Fig. 4. Figure 4a shows the changes of the total Cr and Cr (VI) concentrations after treatment with hollow PANI micro/nanospheres at different pH values, respectively. The Cr (VI) removal capacities of the hollow PANI micro/nanospheres in different pH value can be calculated using the Eq. (1). The corresponding relationship between Cr (VI) removal capacities and pH values is calculated from the values in Fig. 4a by Eq. (1) and shown in Fig. 4b. The obvious decrease of Cr (VI) concentration as the decrease of pH values from 12 to 1 displays that the corresponding Cr (VI) removal capacities of the hollow PANI micro/nanospheres increase with increase of acidity. Especially, the Cr (VI) removal capacity shows a rapid increase when pH is lower than 4. However, the total Cr concentration shows abnormal performance when pH value is below 2, which indicates that the concentration of the Cr (III) sharply increases in the solution. It was reported that, at lower pH (pH below 2), the reduced Cr (III) dominantly existed in Cr^3+^ form, and the literature reported that the protonation extent of the used PANI hollow micro/nanospheres rapidly increased with pH decrease in acidic pH 1–2 [26, 27]. Therefore, the above experiment results can be attributed to that the electrostatic repulsion increases between the used hollow PANI microspheres and the reduced Cr (III), which overcomes chelation interaction between them, so as to Cr (III) enter into solution from the surface of PANI, when pH is below 2. When pH is higher than 2, the change tendency of the total Cr concentration is similar to that of Cr (VI) (Fig. 4a), indicating that most of the reduced Cr (III) is removed from the solution. It affirms that the hollow PANI micro/nanospheres are a good candidate for Cr (VI) removal when the pH value between 2 to 4.

**Fig. 5:**
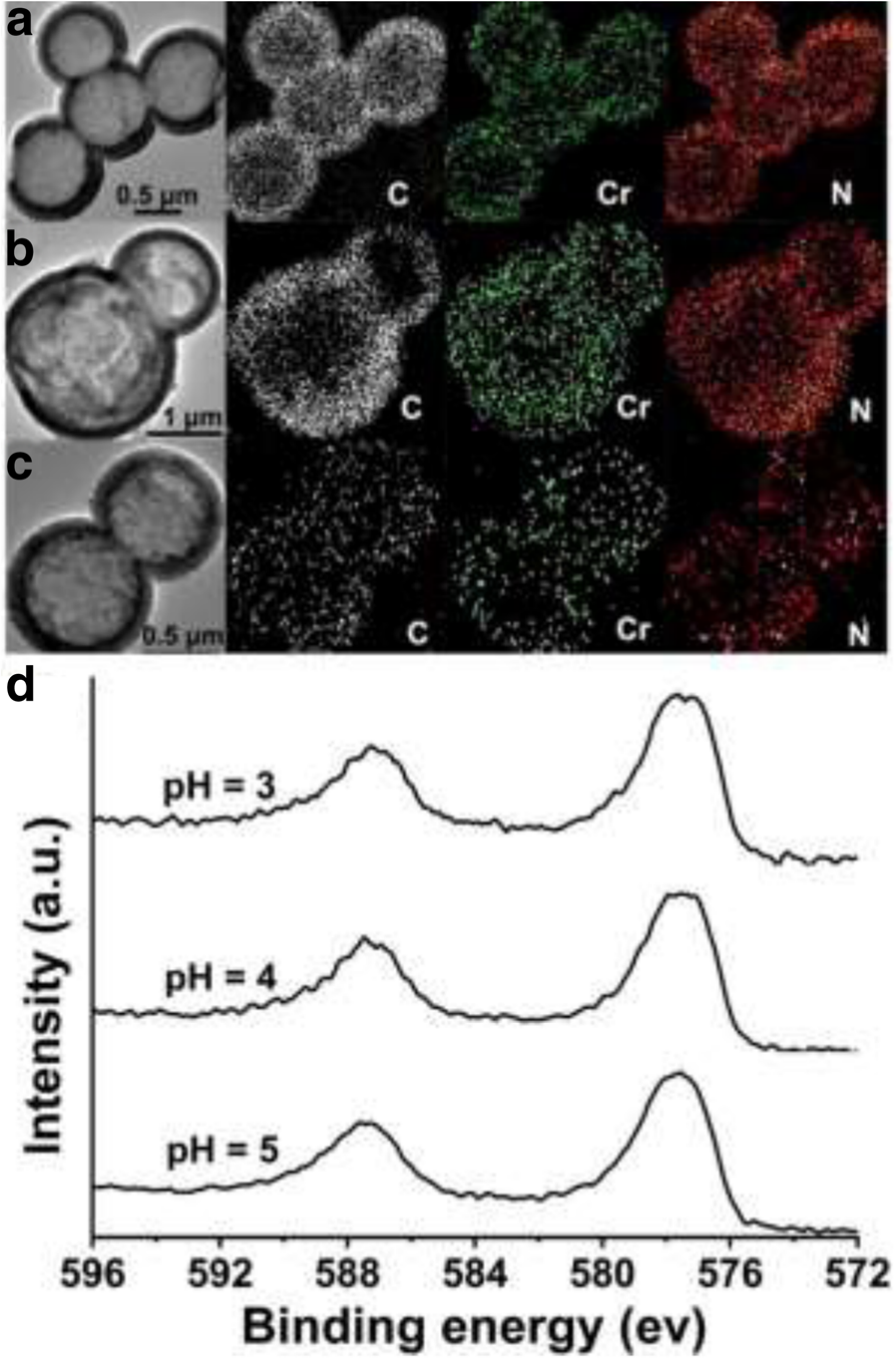
**a**–**c** Element mapping and **d** Cr 2p XPS spectra of the hollow PANI micro/nanospheres after reaction with Cr (VI) (initial Cr (VI) concentration: 1.2 mmol/L (62.4 mg/L); solution pH respectively at 3, 4, and 5)

The hollow PANI microspheres after reaction with Cr (VI) solution at pH 3, 4 and 5 are further investigated. Figure 5a–c shows the element mapping analysis of the used hollow PANI micro/nanospheres, in which Cr element was observed besides C and N elements. It directly demonstrates that the Cr ions were adsorbed indeed by PANI hollow micro/nanospheres. Figure 5d shows the XPS spectrums of the used PANI hollow microspheres at pH 3, 4, and 5, respectively, which exhibits the binding energy of the Cr 2p. In the XPS spectra, two peaks can be observed; the former corresponds to 2p_1/2_, the later to 2p_3/2_. Comparing the three XPS spectrum lines, the binding energy of the Cr 2p_3/2_ locates at 577.4 eV and nothing to do with pH value. It was reported that the bands at 577.4 eV can be attributed to Cr (III) by analogy with other chromium compounds [12, 28]. Thus, the adsorbed Cr are all mainly in Cr (III) form.

Based on all of these results, it can summarize the removal mechanism of Cr (VI) with hollow PANI micro/nanospheres as follows: the Cr (VI) is absorbed on the surface of hollow pristine PANI micro/nanospheres (EB). Then the absorbed Cr (VI) is all reduced to Cr (III). Meanwhile, pristine PANI (EB) is oxidized to pernigraniline form (PB). Cr (VI) in solution exists in the form of acid chromate ion (HCrO_4_^−^) within the pH range (2–6) [25]. At this pH range, a portion of EB PANI is protonated, and the amine group (-NH-) of its molecules exists as amine group (-NH_2_^+^-). Thus, Cr (VI) adsorbed by hollow PANI micro/nanospheres accomplishes through the electrostatic interaction between positive charge PANI and negative charge HCrO_4_^−^. The zeta potential and particle size of the samples were measured with Zetasizer 3000HSa, and the results show that the zeta potential is 38.6 mV and 32.9 mV, and the particle size is about 990 nm and 630 nm for PANI before and after Cr (VI) removal at pH 3, respectively. It means that PANI of before and after Cr (VI) removal can exist stably in the solution. Comparing the molecular structure of EB and PB PANI, it can be found that the solvation of EB PANI of aromatic secondary amine is greater than that of PB PANI of aromatic tertiary amine, because the secondary amine carries one more hydrogen atom than the tertiary amine [29]. Thus, the apparent particle size of EB PANI is greater than that of PB PANI.

**Fig. 6:**
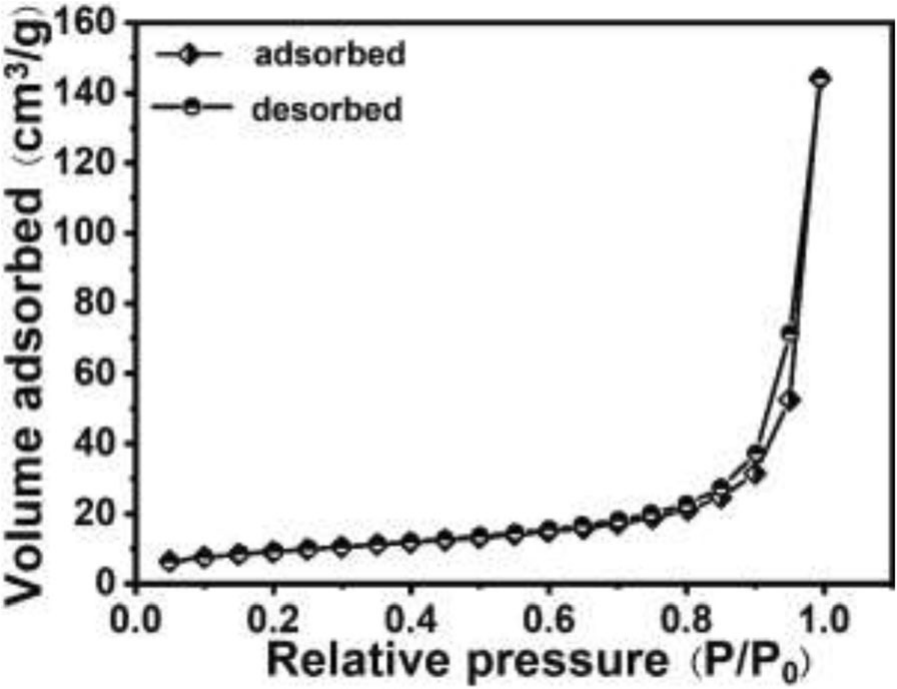
Nitrogen adsorption-desorption isotherms of the as-synthesized hollow PANI micro/nanospheres

As we known, the removal capacity of Cr (VI) mainly depends on the specific surface area of hollow PANI micro/nanospheres. The specific surface area of hollow PANI micro/nanospheres can be obtained by nitrogen adsorption-desorption analysis (shown in Fig. 6), and the BET surface area can be calculated to be 32.813 m^2^/g, indicating higher specific surface area of hollow PANI micro/nanospheres.

**Fig. 7:**
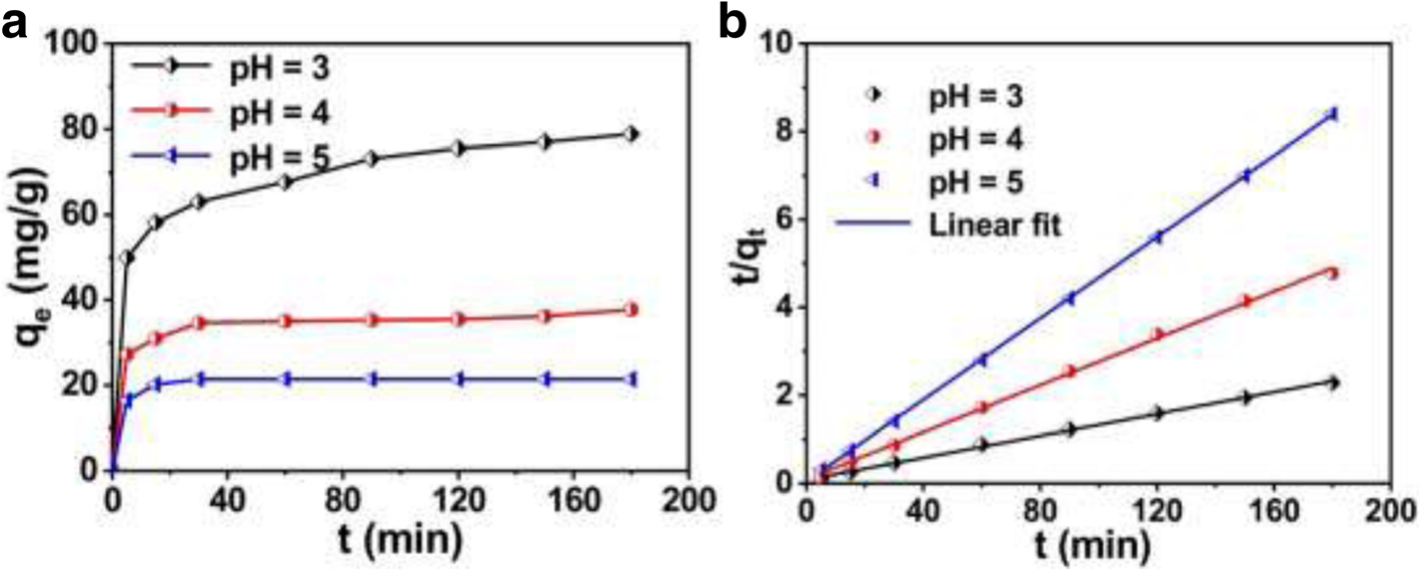
**a** Changes of Cr (VI) concentration at different times (initial Cr (VI) concentration: 1.2 mmol/L (62.4 mg/L), solution pH respectively at 3, 4, and 5), and **b** the pseudo-second-order models on removal kinetic of Cr (VI) with hollow PANI micro/nanospheres

Figure 7a shows the relationship between the removal capacity *q*_t_ of Cr (VI) and the time *t* for solution with different pH values. The removal capacity increases rapidly in the initial stage (0~5 min), and then continues to increase slowly until equilibrium after about 120 min. It indicates that the initial removal occurs on the surface of hollow PANI micro/nanospheres and then proceeds into the inner part [25].

In order to explore the removal Kinetics of Cr (VI) with hollow PANI micro/nanospheres, some models containing the pseudo-first-order and pseudo-second-order models are employed to interpret the experiment data. Here, the pseudo-second-order is the most appropriate model to fit the experimental data, comparing the experiment data with these theoretical models. Figure 7b shows the theoretical Kinetic plots according to the pseudo-second-order Eq. (2). The result shows that *t*/*q*_t_ versus *t* were linear and the correlation coefficients *R*^2^ correspond to 0.99788, 0.99817, and 0.99994, for pH 3, 4 and 5, respectively. In addition, the values of *q*_e_ can be calculated from the slope, i.e., 80.654, 37.48, and 21.56 mg/g corresponding to pH value of 3, 4, and 5, respectively, which are close to the experimental values shown in Fig. 7a.

**Fig. 8:**
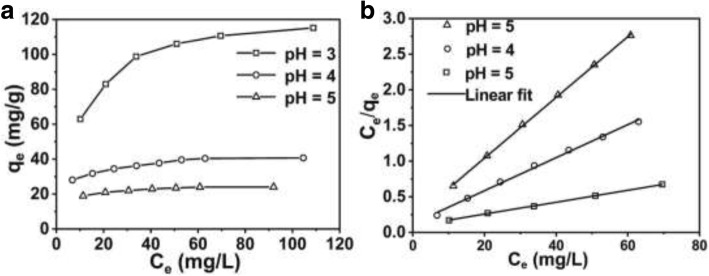
**a** The removal capacity of Cr (VI) at different equilibrium concentration (solution pH respectively at 3, 4, and 5), and **b** corresponding Langmuir removal isotherm model

The Cr (VI) removal capacities of the PANI hollow micro/nanospheres are also related to Cr (VI) concentration. So it is important to investigate the effect of Cr (VI) concentration on the removal capacities of PANI hollow micro/nanospheres. The experiment was carried out at pH 3, 4, and 5, respectively, with various initial concentrations of Cr (VI). Figure 8a shows the changes of the Cr (VI) removal capacity (*q*_e_ (mg/g)) versus equilibrium concentration (*c*_e_ (mg/L)). As shown in Fig. 8a, the removal capacity increases faster at lower Cr (VI) concentration and tend to be a constant value at higher concentration, that is, maximum Cr (VI) removal capacity of hollow PANI micro/nanospheres is achieved. To describe the experimental result of Cr (VI) removal isotherm, three important isotherms models, which are Langmuir, Freundlich, and Temkin models, are selected. However, only the Langmuir model can fit the experimental data. Figure 8b shows the Langmuir plots for the removal of Cr (VI) by hollow PANI micro/nanospheres the Langmuir Eq. (3). The result shows that *c*_e_/*q*_e_ versus *c*_e_ is linear at pH 3, 4, and 5, respectively (correlation coefficient *R*^2^ = 0.99950, 0.99875, and 0.99962 at pH 3, 4, and 5). In addition, the values of *q*_m_ which is calculated from the slope are 25.61, 43.20, and 127.88 mg/g at pH 5, 4, and 3, respectively, which are close to the experimental values shown in Fig. 8a. The Langmuir isotherm is based on assumption of no interaction between homogeneous structure adsorbent and monolayer coverage. The removal of Cr (VI) from the solution on the hollow PANI micro/nanosphere matches with the monolayer mode. It can affirm that no further adsorption can take place when active sites on the surface of the hollow PANI micro/nanospheres are occupied by Cr (VI).

The comparison of the maximum Cr(VI) removal capacity of the hollow PANI micro/nanospheres synthesized in this study with that reported in the literatures is shown in Table 1. It can be seen that the polyaniline hollow micro/nanospheres exhibits higher Cr(VI) removal capacity than that of many other removers. These results suggest that the hollow PANI micro/nanospheres can be considered as a promising material for the removal of Cr(VI) from aqueous solution.

**Table 1:** Comparison of the maximum removal capacity of Cr (VI) on various removers

Removers	pH	*q*_m_ (mg g^−1^)	References
Hollow PANI spheres	3.0	127.88	This work
Hollow PANI spheres	4.0	43.20	This work
Hollow PANI spheres	5.0	25.61	This work
Poly(o-toluidine)/pumice	3.0	19.4	[30]
Hazelnut shell activated carbon (H_2_SO_4_)	3.0	52.2	[31]
PANI-jute	3.0	62.9	[32]
Waste Weed, Salvinia cucullata	4.9	23.98	[33]
Water lily	5.0	5.11	[34]
PANI nanowire/tubes	5.0	86.7	26
Wood activated carbon	2.0–5.0	29.9–26.6	[35]

**Fig. 9:**
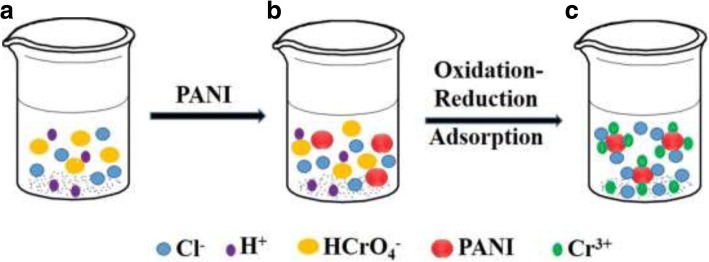
The schematic diagram of Cr (VI) removal. **a** Wastewater containing Cr (VI) under acidic conditions, **b** adding PANI to wastewater containing Cr (VI), and **c** wastewater of Cr (VI) removal

On the basis of the aforementioned results, the process of Cr (VI) removal can be explained as following. The initial solution contains Cl^−^, H^+^, HCrO_4_^−^ ions, and so on (Fig. 9a). Under the acid medium condition, emeraldine oxidation state of PANI (EB) and Cr (VI) ions are oxidized and reduced to pernigraniline state (PB) and Cr^3+^ ions, respectively (Fig. 9b). The main reaction proceeding can be demonstrated as follows under acid solution [36]:

4 $$ 3\mathrm{PANI}\ \left(\mathrm{EB}\right)+6{\mathrm{Cl}}^{\hbox{-} 1}\hbox{-} 6{\mathrm{e}}^{\hbox{-} 1}\to 3\mathrm{PANI}{\left(\mathrm{Cl}\right)}_2\left(\mathrm{PB}\right) $$

5 $$ 2{{\mathrm{H}\mathrm{CrO}}_4}^{\hbox{-} 1}\kern0.5em +14{\mathrm{H}}^{+}\kern0.5em +6{\mathrm{e}}^{\hbox{-} 1}\kern0.5em \to 2{\mathrm{Cr}}^{3+}\kern0.5em +\kern0.5em 8{\mathrm{H}}_2\mathrm{O} $$

This reaction occurs simultaneously on the surface of PANI. The resulting Cr^3+^ ions are absorbed on the surface of PANI micro/nanospheres (Fig. 9c). As mentioned above, PANI micro/nanospheres are hollow spheres, and most hollow spheres have many holes, so the outer and inner surface of PANI micro/nanospheres can absorb lots of Cr^3+^ ions because of the hollow structure (Fig. 1).

**Scheme 1:**
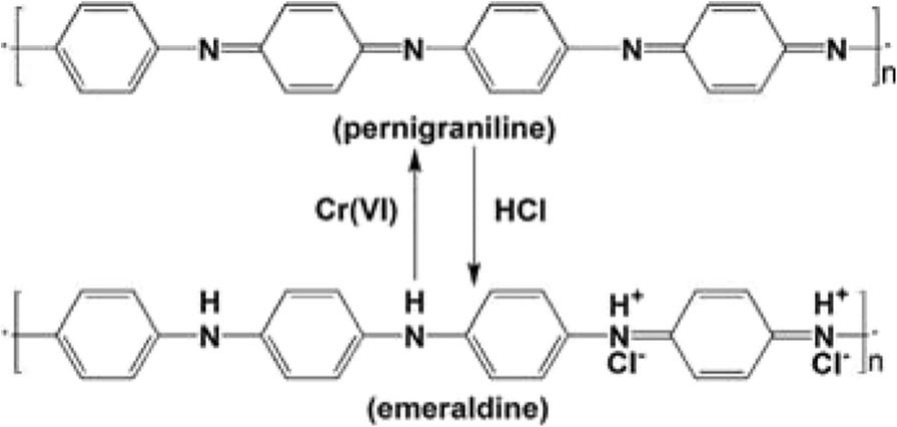
Schematic illustration the conversion between pernigraniline and emeraldine of PANI

It was reported that the pernigraniline is unstable under ambient conditions and easily reduced to emeraldine oxidation state in strong acid solution, such as HCl and H_2_SO_4_ [26]. This indicates that the used hollow PANI micro/nanospheres can be easily regenerated through acid treatment. The conversion between the pernigraniline and the emeraldine state can be shown as Scheme 1. For example, the used hollow PANI micro/nanoshpheres are further treated with 1 M HCl for 0.5 h and then reused for Cr (VI) removal. It can be found from Table 2 that the removal capacity of the first reused hollow PANI micro/nanoshpheres is probably close to the initial PANI removal capacity [26, 37]. It can be concluded that the hollow PANI micro/nanospheres are a reproducible material for Cr (VI) removal.

**Table 2:** Comparison of Cr (VI) removal capacity at equilibrium treated by hollow PANI micro/nanospheres before and after regeneration

	*q*_e_ (the initial PANI) (mg g^−1^)	*q*_e_ (the 1st regeneration) (mg g^−1^)	*q*_e_ (the 2nd regeneration) (mg g^−1^)
pH = 3	78.88	78.84	59.87
pH = 4	37.73	37.73	31.66
pH = 5	21.43	21.43	12.84

## Conclusions

A large quantity of hollow PANI micro/nanospheres has been fabricated by a simple polymerization of monomer in alkaline solution with Triton X-100 Micelles as templates. The hollow micro/nanospheres can rapidly and effectively remove Cr (VI) in a wide pH range. The removal kinetics data is fitted to pseudo-second-order model well and the Cr (VI) removal isotherm can be described by Langmuir isotherm model. The maximum removal capacity of hollow PANI micro/nanospheres can reach 127.88 mg/g at pH 3. Moreover, the used hollow PANI micro/nanospheres can be easily regenerated by means of treatment with acid solution, maintaining roughly the same Cr (VI) removal capacity. The present work indicates that the hollow PANI micro/nanospheres are an effective and reproducible material for removal toxic Cr (VI) from wastewater.

## Abbreviations

APS: Ammonium persulfate
B: Benzenoid ring
EB: Emeraldine
FESEM: Field-emission scanning electron microscope
FTIR: Fourier-transform infrared spectroscopy
ICP: Inductively coupled plasma emission spectrometer
LB: Leucoemeraldine
PANI: Polyaniline
PB: Pernigraniline
Q: Quinoid ring
TEM: Transmission electron microscope
XPS: X-ray photoelectron spectroscopy
XRD: X-ray diffraction
